# Transcriptome Dynamics and Regulatory Networks of Postnatal Muscle Development in Leizhou Black Goats

**DOI:** 10.3390/ijms27010088

**Published:** 2025-12-21

**Authors:** Jiancheng Han, Jing Huang, Mengning Xu, Yuelang Zhang, Ke Wang, Hanlin Zhou

**Affiliations:** 1 Zhanjiang Experimental Station, Chinese Academy of Tropical Agricultural Sciences, Zhanjiang 524013, China; hanjiancheng810@163.com; 2 College of Animal Science and Technology, Guangxi University, Nanning 530004, China; huangjing07182024@163.com; 3 Sanya Institute, Nanjing Agricultural University, Sanya 572024, China; xmn07210955@163.com; 4 Hainan Institute, Zhejiang University, Sanya 572024, China; zhangyuelang@zju.edu.cn; 5 Sanya Research Institute, Chinese Academy of Tropical Agricultural Sciences, Sanya 572024, China

**Keywords:** Leizhou black goat, muscle development, transcriptome analysis, molecular breeding

## Abstract

Postnatal muscle development involves complex transcriptional regulation that remains poorly characterized in goats. This study employed RNA-Seq to profile the Longissimus dorsitranscriptome of Leizhou Black goats across three developmental stages: birth, six months, and two years. We identified dynamic gene expression patterns, widespread alternative splicing events, and stage-specific co-expression networks that collectively orchestrate muscle maturation. A significant transcriptional shift occurred between six months and two years, marked by the downregulation of proliferation-related genes (e.g., *RRM2*, *TOP2A*) and the activation of pathways governing muscle contraction and energy metabolism. Functional enrichment analyses highlighted the importance of PI3K-Akt, PPAR, and calcium signaling pathways throughout development. Additionally, 905 novel transcripts were discovered, many enriched in mitochondrial functions, indicating incompleteness in the current goat genome annotation. Weighted gene co-expression network analysis revealed modules correlated with developmental stages, and protein–protein interaction analysis identified hub genes regulating cell cycle progression and muscle function. Key results were validated via qRT-PCR, confirming the temporal expression patterns of genes such as *CYP4B1*, *HACD1*, and *ACTC1*. These findings provide mechanistic insights into the transcriptional reprogramming driving postnatal muscle development and offer valuable genetic resources for improving meat production in goats through molecular breeding.

## 1. Introduction

Goat husbandry, concentrated in tropical and semi-arid regions of developing countries, serves as a key income source for smallholders within mixed farming systems [1]. Compared to cattle and monogastric livestock, goats offer higher economic viability due to lower investment costs, faster reproduction rates, diversified products (meat, milk, fiber), efficient use of agricultural byproducts, and strong resilience to drought and heat stress [2]. Furthermore, goat meat is recognized as a nutritious red meat option with low cholesterol and fat content, aligning with modern health-conscious consumer demands [3]. Among the diverse goat genetic resources, the Leizhou Black Goat (LZBG), an indigenous breed native to tropical China, stands out for its remarkable thermo-hygric adaptability, coupled with superior production traits including enhanced reproductive performance, and premium meat quality characteristics [4,5].

However, despite these advantages, the LZBG breed is characterized by a smaller body size compared to commercial breeds [6]. A significant challenge is that their postnatal growth reaches a plateau as early as 8 to 10 months of age, followed by markedly slow development until physical maturity [4]. This prolonged fattening period severely undermines breeding profitability after sexual maturity, prompting many farmers to opt for early mating and slaughter before the goats reach full maturity. These practices, in turn, substantially reduce the overall lambing rate and population size, while also hindering the implementation of scientific breeding programs [5,7]. Consequently, the development of the entire LZBG industry is constrained. To break this bottleneck, it is crucial to elucidate the unique growth patterns of LZBG and identify the functional genes that regulate muscle development across its various stages.

The advent of high-throughput RNA sequencing (RNA-Seq) has revolutionized biological research by enabling comprehensive and unbiased profiling of transcriptomes [8]. This technology offers extensive coverage, powerful analytical capabilities, strong reproducibility, and increasingly low cost. Over the past decade, the application of RNA-Seq has exploded across various fields, including human medicine and developmental biology. Its adoption in livestock research has also become widespread [9]. Notably, the annual number of published transcriptomic studies in livestock has nearly tripled, reflecting its critical role in deciphering the genetic basis of complex economic traits [10]. Moreover, while most transcriptomic research in livestock has focused on reproduction, lactation, and disease, studies targeting growth and developmental traits in goats, particularly during postnatal stages, are relatively scarce compared to species like pigs [11,12]. This highlights a significant knowledge gap in the molecular mechanisms governing muscle development in goats.

To address this gap, we employed a comparative transcriptomic approach. RNA sequencing was performed on Longissimus dorsimuscle samples from Leizhou Black goats at three critical postnatal developmental time points: birth (0 days, representing the starting point of postnatal life), early growth (6 months, selected to precede the breed’s typical growth plateau occurring at 8–10 months of age), and maturity (2 years, corresponding to the optimal commercial slaughter age of 1.5–2 years in this breed). The primary objectives were: (1) to characterize the dynamic changes in the global gene expression profiles throughout postnatal muscle development in LZBG; (2) to identify key functional genes and regulatory pathways orchestrating muscle growth and maturation; and (3) to elucidate the expression patterns of genes potentially associated with the breed’s unique muscle traits. Our findings are expected to provide valuable genetic background information and candidate gene resources for future molecular marker-assisted selection or gene-editing breeding strategies aimed at enhancing muscle yield and quality in goats.

## 2. Results

### 2.1. Overview of Sequencing Data, Quality Control, and Reference-Based Alignment

A total of 27 samples, comprising 9 biological replicates for each of the three developmental stages (0 days, 6 months, and 2 years postpartum), were subjected to RNA sequencing. This yielded an average of 45 million paired-end reads per sample. Following stringent quality control with fastp, high-quality clean data were obtained, with Q30 scores exceeding 97% and GC content consistently around 52% for all samples (Table S1). The clean reads were subsequently aligned to the goat reference genome (ARS1) using HISAT2, achieving an average mapping rate of 94.5%. The overwhelming majority of reads (85–91%) mapped to exonic regions, confirming highly effective mRNA enrichment. The remaining reads mapped to intronic (5–10%) and intergenic regions (3–6%), reflecting the inherent complexity of the transcriptome (Table S2). These results collectively demonstrate the high quality and reliability of the sequencing data, providing a robust foundation for all downstream analyses.

### 2.2. Transcriptomic Landscape and Sample Relationships

**Figure 1:**
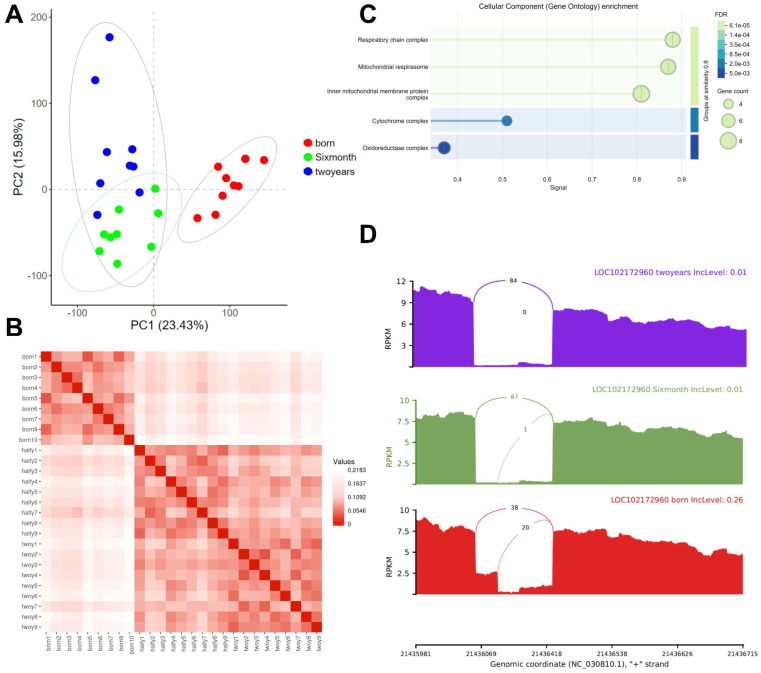
Multi-dimensional transcriptomic analysis of Longissimus dorsimuscle across three developmental stages in Leizhou Black Goats. (**A**) Principal component analysis (PCA) of gene expression profiles based on TPM-normalized values. Samples cluster distinctly by developmental stage: born (red), six-month (green), and two-years (blue). (**B**) Correlation heatmap of gene expression across samples. Red indicates high pairwise correlation coefficients, demonstrating strong intra-group similarity and inter-group divergence. (**C**) Gene Ontology (GO) enrichment analysis of novel transcripts. (**D**) Expression profile and genomic structure of *LOC102172960* across developmental stages. The sashimi plot displays read coverage and splicing patterns, while bar plots (right) show its expression levels (in RPKM) at birth, six-month, and two-years stages.

Principal Component Analysis (PCA) revealed distinct transcriptomic profiles clearly separating the three developmental stages (Figure 1A). The first two principal components, PC1 and PC2, explained 23.43% and 15.98% of the total transcriptional variance, respectively. Samples clustered tightly according to their developmental group, with each stage—birth, six months, and two years—occupying a distinct area on the PCA plot. Notably, the two-year samples exhibited greater dispersion along the PC2 axis, suggesting increased transcriptional heterogeneity in adulthood, with some individuals showing a transcriptional profile closer to the six-month group. This pronounced inter-group separation establishes developmental stage as a primary driver of global gene expression patterns in the Longissimus dorsimuscle of Leizhou Black Goats. Consistently, a correlation heatmap demonstrated strong intra-group similarities, where samples within the same age group showed higher correlation coefficients than those between different groups (Figure 1B).

### 2.3. Identification of Novel Transcripts and Alternative Splicing Events

De novotranscriptome assembly using StringTie identified 905 novel transcripts not previously annotated in the reference genome. Prediction of open reading frames using TransDecoder v5.7.1 indicated that 654 of these novel transcripts are potentially functional. Annotation of the novel transcripts with eggNOG-mapper revealed that 226 could be mapped to mammalian sequences, with 222 receiving putative functional annotations (Table S3). Functional enrichment analysis indicated that these novel transcripts are significantly associated with mitochondrial functions, such as “Respiratory chain complex” and “Inner mitochondrial membrane protein complex” (Figure 1C). Concurrently, rMATS analysis identified 4231 significant alternative splicing (AS) events (FDR < 0.05) across development, with skipped exon (SE) being the most prevalent type. Notably, one Alternative 5′ splice site (A5SS) event showed exceptionally significant differences across the three developmental stages (Figure 1D).

### 2.4. Transcriptomic Dynamics and Functional Shifts During Postnatal Development

**Figure 2:**
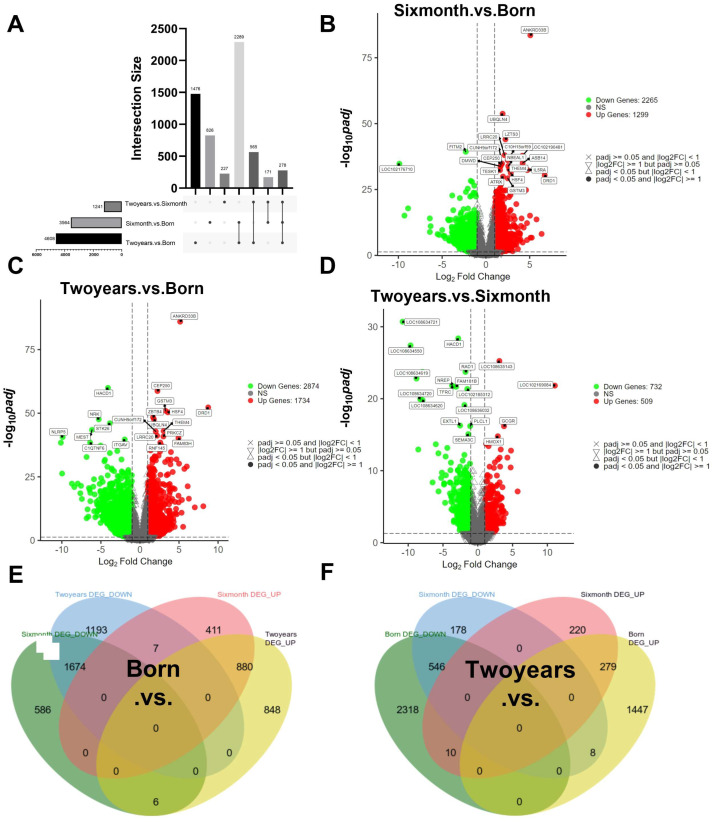
Distribution of Differentially expressed genes across developmental stage comparisons in Leizhou Black Goats. (**A**) Comparative analysis of intersection sizes and data distribution across developmental stage comparisons. Volcano plots displaying genome-wide expression changes for the (**B**) 6 Months vs. Birth, (**C**) 2 Years vs. Birth, and (**D**) 2 Years vs. 6 Months. Venn diagrams summarizing the overlap of up-regulated (**E**) and down-regulated (**F**) DEGs across developmental comparisons.

Analysis of Differentially Expressed Genes (DEGs) revealed specific distribution patterns among the three pairwise comparisons (Figure 2A). A total of 4608 DEGs were identified in the comparison spanning the longest interval (2-year vs. birth), followed by 3564 DEGs between the 6-month and birth stages. Notably, 2289 of these DEGs were common to both comparisons, indicating substantial transcriptomic reprogramming during maturation. Volcano plots illustrated the extensive gene expression changes (Figure 2B–D). Specifically, comparisons against the birth stage showed widespread gene activation and suppression at both six months and two years. In contrast, the two-year versus six-month comparison, while still identifying a substantial number of DEGs (1241), indicated a reduction in the scale of transcriptional changes compared to earlier stages. This phase was characterized by a distinct set of significantly downregulated genes with age yet also included the concerted induction of genes critical for muscle functional maturation, pointing to more refined transcriptional dynamics in adulthood. Further analysis of expression trends revealed that, compared to birth, 1674 DEGs were consistently upregulated and 880 were consistently downregulated at both six months and two years, with only a minimal number of genes (*n* = 7) showing opposite expression trends between these later stages (Figure 2E). A similar consistent up- or down-regulation pattern was observed for DEGs when comparing the two-year stage to the other stages (Figure 2F). Functional annotation of the top 20 DEGs from each pairwise comparison revealed distinct stage-associated biological themes. The top DEGs identified in the 0-day group, when compared to both the 6-month and 2-year groups, were predominantly enriched in functions related to “cell activation involved in immune response” and “morphogenesis of various tissues and systems.” In contrast, the top DEGs derived from the comparison between the 2-year and 6-month groups were significantly enriched in processes such as “muscle structure development” and “skeletal system development” (Supplementary Files S1).

**Figure 3:**
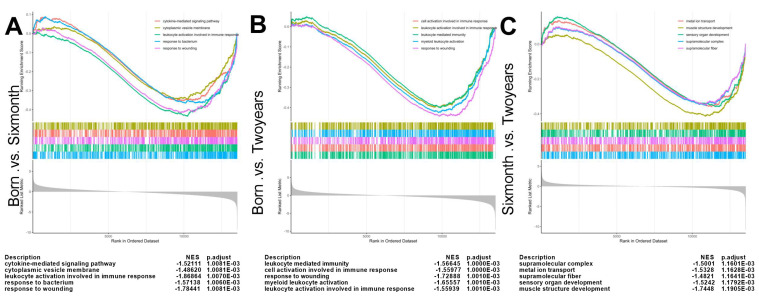
Gene Set Enrichment Analysis reveals stage-specific biological pathways during postnatal muscle development. (**A**) GSEA plot for the comparison Born vs. Six month. (**B**) GSEA plot for the comparison Born vs. Two years. (**C**) GSEA plot for the comparison Six month vs. Two years. The Normalized Enrichment Score and the adjusted *p*-value (padj) for each plotted gene set are indicated directly on the panels.

Functional enrichment of DEGs was performed using both Over-Representation Analysis (ORA) and Gene Set Enrichment Analysis (GSEA). In the birth versus six-month comparison, gene sets related to “immune system process,” “leukocyte activation,” and “cytokine-mediated signaling pathway” were significantly enriched in the six-month group, indicative of active immune signaling during rapid growth (Figure 3A). The birth versus two-year comparison showed enrichment for terms like “cellular component organization” and “extracellular matrix organization,” reflecting structural maturation (Figure 3B). The most pronounced enrichment was observed in the six-month versus two-year comparison, characterized by activation of “myofiber contraction” and “muscle system process” pathways, signifying the presence of extensive muscle development regulation between these two time points (Figure 3C). KEGG pathway analysis highlighted the importance of the PI3K-Akt, PPAR, and Calcium signaling pathways throughout development (Supplementary Files S2).

### 2.5. Co-Expression Network Analysis Identifies Stage-Specific Modules and Key Regulatory Hubs

**Figure 4:**
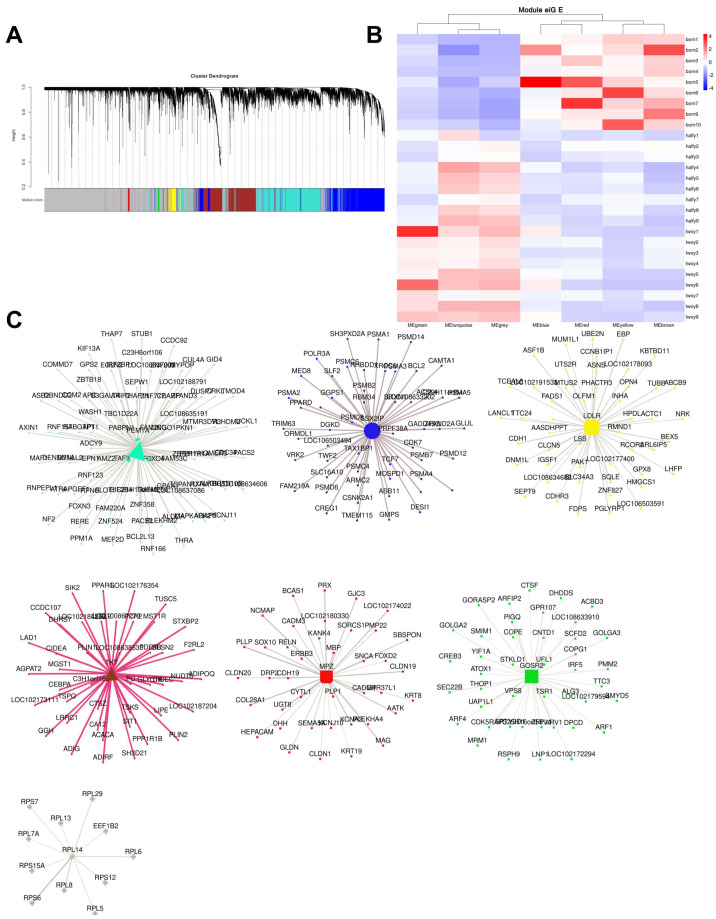
Multiscale transcriptomic network analysis identifies co-expression modules and hub genes during muscle development. (**A**) Cluster dendrogram of genes based on topological overlap matrix (TOM) dissimilarity. Each leaf in the tree represents one gene. Major co-expression modules are assigned different colors below the dendrogram; genes in the gray module are not assigned to any specific module. (**B**) Heatmap showing the adjacency relationships between genes based on TOM. Rows and columns represent gene sets, and samples, the color intensity indicates the strength of co-expression. The side bars represent module membership. (**C**) Protein–protein interaction (PPI) networks of hub genes within key modules. Nodes represent proteins, edges represent interactions, and node size reflects the degree of connectivity. Hub genes with high intramodular connectivity are labeled.

Weighted Gene Co-expression Network Analysis (WGCNA) identified 7 distinct co-expression modules (Figure 4A). The ‘Brown’ module (2008 genes), highly expressed at birth, was enriched for terms like “Extracellular matrix organization” (Figure 4B). The ‘Turquoise’ (2900 genes) and ‘Grey’ (5872 genes) modules, more highly expressed at six months and two years, were enriched for “Regulation of macromolecule biosynthetic process” and “Mitochondrial respiratory chain complex assembly,” respectively (see Supplementary Materials). Key hub genes within these modules included *LDLR* (blue), *GOSR2* (green), *TKT* (brown), *RPL14* (grey), *FEM1A* (turquoise), *MPZ* (red), and *SSX2IP* (yellow) (Figure 4C).

Protein–Protein Interaction (PPI) network analysis of stage-specific DEGs, integrated with WGCNA hub genes, identified 11 central regulators (degree > 20). These included core cell proliferation regulators (*RRM2*, *TOP2A*, *BUB1B*), essential factors for mitotic spindle assembly and chromosome segregation (*DLGAP5*, *KIF11*, *CDCA8*), and established markers of cell cycle progression (*CKS2*, *MKI67*, *SPAG5*, *ARHGAP11A*, *KIAA0101*). This underscores their collective importance in supporting the high rates of myonuclear accretion and hyperplastic growth characteristic of the early postnatal phase (Supplementary Files S3).

### 2.6. Temporal Expression Patterns of Key DEGs Validated by qRT-PCR

**Figure 5:**
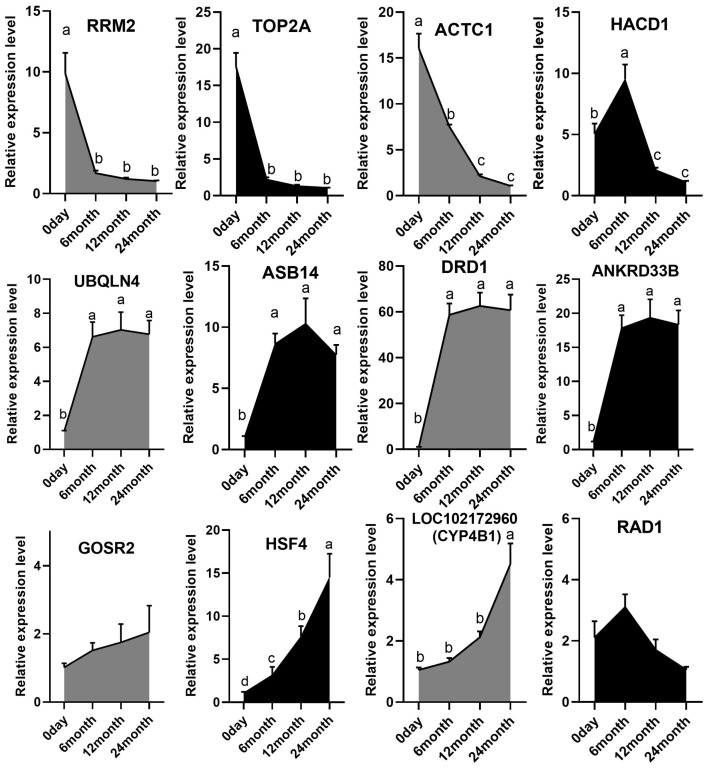
Validation of key differentially expressed genes by qRT-PCR across four developmental stages. Temporal expression patterns of twelve candidate genes were analyzed by quantitative real-time PCR in Longissimus dorsimuscle samples collected from Leizhou Black goats at 0 day, 6 months, 12 months, and 24 months of age. Data are presented as mean ± SEM (*n* = 4 × 12 = 48). Different lowercase letters (a–d) above bars indicate statistically significant differences (*p* < 0.05) as determined by one-way ANOVA followed by Tukey’s post hoc test.

To prioritize key genes for experimental validation, we employed an integrative multi-analysis consensus approach (Table 1). Candidate genes were first compiled from several independent, high-confidence sources: the most significantly differentially expressed genes (DEGs) from all pairwise developmental comparisons, the central regulatory genes identified in the protein–protein interaction (PPI) network, the hub genes derived from weighted gene co-expression network analysis (WGCNA), and the DEG of primary interest from alternative splicing analysis. From this comprehensive candidate pool, we prioritized genes that were recurrently identified across multiple independent analyses. Subsequently, 1–2 additional DEGs were representatively selected from each independent analytical category. This process resulted in 12 high-confidence candidate genes, integrating transcriptional, co-expression, and protein interaction evidence. The expression profiles of twelve selected DEGs were rigorously validated across four developmental stages (0 day, 6 months, 12 months, and 24 months) using qRT-PCR. The results confirmed distinct, stage-specific expression patterns that were largely consistent with and extended the findings from the RNA-Seq analysis (Figure 5). Genes functionally associated with cell proliferation, such as *RRM2* and *TOP2A*, displayed their highest expression levels at birth, followed by a significant and progressive decline throughout postnatal development, indicative of a transition from hyperplastic growth to functional maturation. Conversely, genes implicated in muscle structure and functional development, including *ACTC1* and *HACD1*, exhibited a more complex expression dynamics, characterized by an initial increase that peaked during the rapid growth phase (6 or 12 months), followed by a subsequent decrease. Furthermore, genes such as *UBQLN4* and *ASB14* maintained relatively stable and high expression levels across multiple time points. Notably, *HSF4* and *LOC102172960* demonstrated a marked upward trend in expression, suggesting their potential roles in later-stage muscle maturation or stress adaptation. But the expression of *GOSR2* and *RAD1* did not show statistically significant variations across the stages by qRT-PCR, which was not entirely consistent with the RNA-Seq predictions, highlighting the importance of experimental validation in transcriptomic studies (Table S5).

## 3. Discussion

Postnatal muscle development is a complex biological process orchestrated by precise transcriptional regulation. This study provides a comprehensive transcriptomic profile of the Longissimus dorsimuscle across three critical developmental stages in Leizhou Black goats. While our analysis primarily focused on gene-level expression patterns to identify stage-specific regulators and network-level interactions, we revealed dynamic gene expression patterns, alternative splicing events, and co-expression networks that collectively underlie postnatal muscle development. These molecular features reflect a stage-driven transcriptional reprogramming that facilitates the transition from hyperplastic growth to functional maturation.

Principal component and correlation analyses demonstrated that developmental stage is the primary determinant of transcriptional profiles in muscle tissue, with samples clustering distinctly by age. Increased transcriptional heterogeneity in two-year-old adults may reflect accumulating physiological variations, such as metabolic status, fiber-type composition, and individual responses to environmental factors, which become more pronounced with maturity. We further identified a substantial number of novel transcripts, many enriched in mitochondrial organization and respiratory chain complexes, suggesting that the current goat reference genome annotation remains incomplete, particularly concerning metabolic components critical for muscle energy metabolism. This limitation, along with the challenges in achieving robust transcript-isoform quantification with short-read data, guided our focus toward gene-level analysis. Notably, stage-specific alternative splicing and differential expression of *LOC102172960* (*CYP4B1*), validated by qPCR, highlight this gene as a compelling candidate for future functional studies on muscle development.

Temporal analysis of differential gene expression revealed coordinated transcriptional reprogramming throughout postnatal development. The largest number of DEGs between the two-year and six-month stages indicates that this transition represents the most dramatic phase of transcriptional change, likely corresponding to the shift from rapid growth to metabolic maturation. The minimal number of genes showing reversed expression trends between six months and adulthood suggests that, once established, developmental pathways are generally maintained. Functional enrichment analyses delineated clear biological transitions: early enrichment of immune and inflammatory signaling pathways (birth to six months) may reflect both immune system development and muscle remodeling processes involving satellite cell activation and tissue reorganization. Subsequent enrichment of extracellular matrix and cellular component organization (birth to two years) points to ongoing structural maturation, while the pronounced activation of muscle contraction and energy metabolism pathways between six months and two years signals functional maturation, characterized by enhanced contractile protein expression, calcium handling, and oxidative phosphorylation. The persistent prominence of PI3K-Akt, PPAR, and calcium signaling pathways underscores their fundamental roles in coordinating muscle growth, metabolism, and function. We acknowledge that transcript-isoform level analysis could provide additional biological insights, and future studies employing long-read sequencing technologies would be valuable to explore this dimension of transcriptional regulation.

The WGCNA and PPI network analyses provided additional regulatory insights. The identification of stage-specific modules and hub genes offers a systems-level view of the coordinated gene expression programs driving muscle development. The enrichment of extracellular matrix organization in modules expressed at birth aligns with the importance of the muscle microenvironment for early development. Conversely, the mitochondrial and biosynthetic process enrichment in later stages reflects the increasing metabolic demands of mature muscle. The PPI network highlighted central regulators of cell proliferation and chromosome segregation, emphasizing the importance of precisely controlled nuclear division during the hyperplastic growth phase.

Finally, qPCR validation of ten key DEGs—*CYP4B1*, *RRM2*, *HACD1*, *ACTC1*, *TOP2A*, *ASB14*, *ANKRD33B*, *UBQLN4*, *DRD1*, and *HSF4*—confirmed their dynamic expression patterns and suggested diverse regulatory roles. *RRM2*, a ribonucleotide reductase subunit, has been shown to promote myoblast proliferation while inhibiting differentiation and muscle regeneration in avian models [13], highlighting its critical function in early-phase hyperplastic growth [14]. Similarly, *ASB14*, an E3 ubiquitin ligase component, participates in the ubiquitin–proteasome system by tagging specific proteins for degradation, thereby potentially influencing muscle homeostasis through the regulation of signal transduction and stress response pathways [15,16]. CYP4B1has been implicated in muscle and intramuscular fat development [17], and its knockout suppresses hypertrophy in cardiomyocytes [18]. The *HACD1* (3-Hydroxyacyl-CoA Dehydratase 1) emerges as a pivotal regulator of postnatal muscle development, primarily through its fundamental function in modulating membrane composition and fluidity to facilitate myoblast fusion [19]. Deficiency in *HACD1* function disrupts lipid-dependent growth mechanism, leading to impaired myoblast fusion, myofiber hypotrophy, and generalized muscle weakness [20], which is evidenced in Hacd1-knockout models and is associated with congenital myopathies characterized by fiber size disproportion in both humans and dogs [19,21]. As the predominant actin isoform in adult cardiac muscle and a significant component in skeletal muscle, *ACTC1* provides the mechanical foundation for muscle contraction by polymerizing into thin filaments that interact with myosin [22,23], and has been shown to regulate myoblast proliferation and differentiation in cattle [24]. Emerging evidence links *UBQLN4* to the regulation of mitochondrial quality control and autophagy [25], processes increasingly recognized as vital for muscle homeostasis and adaptation [26]. And the DRD1 gene, encoding dopamine receptor D1, represents a compelling non-canonical regulator of muscle development, potentially linking neural signaling to the regulation of muscle metabolism and functional adaptation [27]. *DRD1* may influence muscle physiology by regulating glucose uptake, glycogen metabolism, and lipid utilization, thereby fine-tuning energy homeostasis in response to dopaminergic stimuli [28]. Although direct mechanistic evidence remains limited for others (*ANKRD33B*, *TOP2A*, *HSF4*), their expression patterns and putative functions suggest involvement in myogenesis [29], cell cycle progression [30], and stress adaptation [31].

This study has several limitations. First, mRNA-level analysis does not capture post-translational modifications or protein activity changes that contribute to muscle development. Second, focusing on a single muscle type limits generalizability, as different muscles may develop via distinct mechanisms. Third, our analysis was conducted at the gene level, and thus did not explore differential expression or splicing at the transcript-isoform level. This decision was influenced by the current limitations in the comprehensiveness and resolution of the goat reference genome annotation, which can affect the robustness of such analyses. Furthermore, the current findings are based on a single breed; comparative transcriptomic studies across different goat breeds in the future would help to more clearly delineate the unique molecular features underlying the developmental physiology of Leizhou Black goats. Future studies integrating proteomics, single-cell sequencing, long-read transcriptome sequencing across multiple muscle types, and cross-breed comparisons will help overcome these limitations and provide deeper mechanistic insights. Functional validation of novel transcripts and genetic candidates also represents an important direction for further research.

## 4. Materials and Methods

### 4.1. Animal Resources, Phenotypic Data Collection, and RNA Sequencing

Purebred Leizhou black goats were raised at the Zhanjiang Hainan Black Goat Breeding Conservation Farm in western Guangdong Province, China. In accordance with the structure of the local production system, where females constitute the primary source of commercial meat animals, this study utilized exclusively female goats. A total of 27 Longissimus dorsimuscle samples were dissected from these female goats, comprising 9 biological replicates for each of the three developmental stages (0 days, 6 months, and 2 years postpartum). Total RNA was extracted from the muscle tissues using Trizol reagent. RNA integrity and concentration were assessed using an Agilent 2100 Bioanalyzer (Agilent Technologies, Santa Clara, CA, USA), and samples with RNA integrity numbers (RIN) greater than 7.0 were used for subsequent library construction. Strand-specific sequencing libraries were prepared and sequenced on the Illumina NovaSeq 6000 platform (Illumina Inc., San Diego, CA, USA) to generate 150 bp paired-end reads. Additionally, an independent set of Longissimus dorsimuscle samples (*n* = 48) was collected from female Leizhou black goats at 0 days, 6 months, 12 months, and 24 months of age (12 biological replicates per time point, distinct from the sequenced samples) for subsequent validation of differentially expressed genes (DEGs).

### 4.2. Data Preprocessing, Quality Control, Read Mapping, and Transcript Assembly

Raw sequencing reads were processed using fastp (v0.23.1) to obtain high-quality clean data [32]. Adapter sequences and low-quality bases were removed using the parameters: --overrepresentation_analysis --trim_front1 2 --trim_front2 2 --cut_front --cut_tail --cut_window_size 3 --cut_mean_quality 30. Quality metrics, including Q20, Q30, and GC content, were assessed for all samples. Clean reads were aligned to the goat reference genome (ARS1.0) using HISAT2 (v2.2.1) with the --dta parameter [33]. The resulting SAM files were converted to sorted BAM files. Alignment statistics, including mapping rate and genomic distribution of reads (exonic, intronic, intergenic), were evaluated using Qualimap (v2.2.2) [34]. Transcript assembly was performed for each sample using StringTie (v2.1.5) [35]. Novel transcripts were identified using gffcompare (v0.12.6) by filtering for intergenic transcripts (class code “u”) with a length ≥ 100 bp and at least two exons. The coding potential of novel transcripts was predicted using TransDecoder (v5.7.1), and functional annotation was performed with eggNOG-mapper (v5.0) [36]. The identification and quantification of alternative splicing (AS) events across developmental stages were performed using rMATS (replicate Multivariate Analysis of Transcript Splicing) software (version 4.1.1) [37].

### 4.3. Gene Expression Quantification and Differential Expression Analysis

Read counts for each gene were generated using featureCounts (v2.0.1) from the Subread package with parameters -p -t exon -g gene_id [38]. Gene expression levels were normalized and reported as FPKM and TPM. Differential expression analysis was performed using DESeq2 (v1.26.0) in R [39]. Genes with an adjusted *p*-value (padj) < 0.05 and an absolute log_2_ fold change (|log_2_FC|) > 1 were considered significantly differentially expressed.

### 4.4. Genetic Variant Calling and Functional Enrichment Analysis

Variant calling (SNPs and InDels) was performed using the Genome Analysis Toolkit (GATK) (v4.2.2.0) best practices workflow [40]. Variants were filtered and functionally annotated using the Variant Effect Predictor (VEP) (v104.3). Functional enrichment analyses, including Gene Ontology (GO) and Kyoto Encyclopedia of Genes and Genomes (KEGG) pathway analyses, were conducted using the clusterProfiler package (v3.14.3) in R. Both Over-Representation Analysis (ORA) for differentially expressed genes and Gene Set Enrichment Analysis (GSEA) using the entire ranked gene list were performed. Terms with a padj < 0.05 were considered significantly enriched. KEGG pathway maps were visualized using the pathview R package. Functional annotation of the top 20 DEGs from each pairwise comparison was performed using the DAVID online database (v2023q4).

### 4.5. Co-Expression and Protein–Protein Interaction Network Analysis

A co-expression network was constructed using the WGCNA package (v1.70.3) in R [41]. A soft-thresholding power was selected based on the scale-free topology criterion. Genes were clustered into modules using hierarchical clustering with a dynamic tree-cutting algorithm. Module eigengenes (MEs) were calculated and correlated with phenotypic traits to identify biologically significant modules. Functional enrichment analysis was performed on genes within key modules. The protein–protein interaction (PPI) network for differentially expressed genes was constructed by querying the STRING database (v10) [42]. The network was filtered to retain interactions with a combined confidence score > 0.7 and visualized using the igraph and networkD3 packages in R. Hub genes were identified based on their connectivity degrees within the network.

### 4.6. Temporal Expression Profiling of Selected DEGs by qRT-PCR

To further validate the temporal expression patterns of key DEGs identified through transcriptomic analyses, a subset of candidate genes (*n* = 12) from the above results was selected for expression profiling across four developmental stages (0 days, 6 months, 12 months, and 24 months) using quantitative real-time PCR (qRT-PCR). Gene-specific primers were designed using NCBI Primer-BLAST (https://www.ncbi.nlm.nih.gov/tools/primer-blast/index.cgi, accessed on 17 December 2025) to span exon–exon junctions (Table S4). *GAPDH* was used as the internal reference gene for normalization. Total RNA from the independent validation cohort (*n* = 48) described in Section 2.1 was reverse-transcribed into cDNA for analysis. Relative expression levels were calculated using the 2^−ΔΔCt^ method. Statistical differences in gene expression across the four developmental time points were assessed using one-way analysis of variance (ANOVA) followed by Tukey’s honestly significant difference post hoc test for multiple comparisons. A *p*-value < 0.05 was considered statistically significant.

## 5. Conclusions

This study delineates a transcriptional reprogramming event during postnatal muscle development in Leizhou Black goats, characterized by a shift from hyperplastic growth to metabolic and functional maturation between six months and two years of age. Through integrative analysis of stage-specific expression patterns, functional annotation of DEGs, and co-expression networks, we identified ten crucial candidate genes (including *RRM2*, *TOP2A*, and *DRD1*) that potentially regulate muscle development. These findings provide valuable insights into the molecular basis of muscle development and offer critical resources for future genetic improvement in goat breeding.

## Figures and Tables

**Table 1:** Key regulatory genes prioritized by multi-analysis consensus.

DEGs Type	Gene Name
Sixmonth.vs.Born_DEG	***ASB14***, *PDK4*, *FBN2*, ***ACTC1***, *TESK1*, *MTUS2*, ***ANKRD33B***, ***UBQLN4***, *FITM2*, *LOC102176710*, ***DRD1***, *CEP250*, ***HSF4***
Twoyears.vs.Born_DEG	*LOC102176710*, ***ANKRD33B***, *CEP250*, *HSF4*, ***HACD1***, *NRK*, ***TOP2A***, *ACTC1*, ***RRM2***, *C1QTNF6*, ***UBQLN4***
Twoyears.vs.Sixmonth_DEG	***HACD1***, *TFRC*, *WISP2*, *CCND1*, *LOC108634721*, *GCGR*, ***RAD1***, *LOC108634619*, *LOC102169084*, *NREP*, *TFRC*
PPI central regulators_DEGs	***RRM2***, ***TOP2A***, *BUB1B*, *CKS2*, *MKI67*, *SPAG5*, *ARHGAP11A*, *KIAA0101*
WGCNA Key hub DEGs	*LDLR*, ***GOSR2***, *TKT*, *RPL14*, *FEM1A*, *MPZ*, *SSX2IP*
Alternative Splicing_DEGs	***LOC102172960*** (***CYP4B1***)

Note: Bold font highlights the 12 DEGs selected for validation of temporal expression patterns by qRT-PCR.

## Data Availability

The original contributions presented in this study are included in the article/Supplementary Materials. Further inquiries can be directed to the corresponding authors.

## Supplementary Materials

The supporting information can be downloaded at: https://www.mdpi.com/article/10.3390/ijms27010088/s1.
