# Supplementary material for: Transcriptome Dynamics and Regulatory Networks of Postnatal Muscle Development in Leizhou Black Goats
Source: Int J Mol Sci. 2025 Dec 21;27(1):88. doi: 10.3390/ijms27010088 (PMC12785553; doi:10.3390/ijms27010088)
Supplement: Supplementary file 1 [file ijms-27-00088-s001.zip › Supplementary Files S2 Functional Enrichment Analysis (GSEA and ORA) of DEGs Across Three Developmental Stages/ORA/KEGG/time_expression_twoyear.vs.born/KEGG_pathview/chx04926.pathview.html]

KEGG PATHWAY: Relaxin signaling pathway - Capra hircus (goat)


# Relaxin signaling pathway - Capra hircus (goat)
