# Supplementary material for: Transcriptome Dynamics and Regulatory Networks of Postnatal Muscle Development in Leizhou Black Goats
Source: Int J Mol Sci. 2025 Dec 21;27(1):88. doi: 10.3390/ijms27010088 (PMC12785553; doi:10.3390/ijms27010088)
Supplement: Supplementary file 1 [file ijms-27-00088-s001.zip › Supplementary Files S3 WGCNA of DEGs Across Three Developmental Stages/2.Network/network.html]

#### 操作网络

节点标签:


字体大小:


节点颜色:


节点大小:


节点形状:


{{ key }}


连线颜色:


连线粗细:


连线形状:


网络布局:


导出图片:


导出


导出网络拓扑参数:


导出
