# Supplementary material for: Transcriptome Dynamics and Regulatory Networks of Postnatal Muscle Development in Leizhou Black Goats
Source: Int J Mol Sci. 2025 Dec 21;27(1):88. doi: 10.3390/ijms27010088 (PMC12785553; doi:10.3390/ijms27010088)
Supplement: Supplementary file 1 [file ijms-27-00088-s001.zip › Table S1,S2 and S4.pdf]

Supplementary Table

Table S1. Summary of sequencing data and alignment statistics.

Sample ID	Total Reads	Total Bases	q20 Rate	q30 Rate	Mapping Rate
born1	62003044	8906306124	0.995914	0.977116	0.945
born2	65727136	9426149440	0.996150	0.978296	0.945
born3	47828066	6859280926	0.996044	0.977236	0.943
born4	52109636	7484462253	0.995986	0.977407	0.946
born5	63876636	9177952820	0.996151	0.978211	0.941
born6	53991012	7775024667	0.996105	0.978168	0.941
born7	55649684	7995816298	0.996055	0.977729	0.937
born9	60440264	8660081184	0.996201	0.978000	0.947
born10	58163120	8297413569	0.996277	0.978979	0.945
halfy1	76659494	10830773870	0.994728	0.969105	0.948
halfy2	55144830	7788022934	0.994682	0.968734	0.949
halfy3	63570184	8941455665	0.994768	0.969028	0.949
halfy4	61008404	8623070410	0.994846	0.969455	0.950
halfy5	51376882	7303526226	0.994794	0.969248	0.937
halfy6	70657192	9966154404	0.994736	0.969133	0.955
halfy7	57808022	8197536346	0.994639	0.967613	0.947
halfy8	59457684	8429298295	0.994785	0.968504	0.944
halfy9	62894982	8875342654	0.994703	0.968417	0.951
twoy1	64168262	9061566228	0.994626	0.967357	0.948
twoy2	77414994	10908201462	0.994922	0.969882	0.950
twoy3	78547018	10999768831	0.994813	0.969791	0.946
twoy4	78670244	11055934490	0.994805	0.969846	0.954
twoy5	53773568	7552928216	0.994663	0.968389	0.946
twoy6	67632122	9499643980	0.994842	0.969645	0.945
twoy7	83021390	11693729172	0.994902	0.969855	0.944
twoy8	64320962	9106101224	0.994871	0.969676	0.942
twoy9	68174090	9638709993	0.994879	0.969770	0.951

Table S2. Genomic distribution of aligned reads.

Sample ID	Exonic	Intronic	Intergenic	Overlapping Exon
born1	47,621,170 (87.97%)	4,362,024 (8.06%)	2,150,153 (3.97%)	1,443,043 (2.67%)
born2	48,843,885 (87.09%)	4,927,808 (8.79%)	2,312,187 (4.12%)	1,406,828 (2.51%)
born3	36,037,225 (87.11%)	3,612,349 (8.73%)	1,719,811 (4.16%)	1,066,653 (2.58%)
born4	39,254,198 (84.81%)	4,987,230 (10.78%)	2,043,479 (4.42%)	1,190,537 (2.57%)
born5	47,705,401 (86.96%)	4,753,546 (8.67%)	2,397,384 (4.37%)	1,463,000 (2.67%)
born6	41,237,050 (89.63%)	3,318,882 (7.21%)	1,453,912 (3.16%)	1,188,998 (2.58%)
born7	40,934,523 (87.75%)	4,118,165 (8.83%)	1,594,441 (3.42%)	1,286,417 (2.76%)
born9	45,896,040 (89.12%)	3,515,222 (6.83%)	2,085,532 (4.05%)	1,258,006 (2.44%)
born10	35,742,543 (86.14%)	4,267,462 (10.28%)	1,484,179 (3.58%)	1,192,040 (2.87%)
halfy1	58,793,934 (88.23%)	5,867,219 (8.81%)	1,972,536 (2.96%)	1,742,439 (2.61%)
halfy2	43,781,890 (89.59%)	3,513,821 (7.19%)	1,573,319 (3.22%)	1,118,593 (2.29%)
halfy3	48,988,318 (88.45%)	4,676,076 (8.44%)	1,717,995 (3.1%)	1,451,820 (2.62%)
halfy4	48,569,842 (91.22%)	3,254,894 (6.11%)	1,419,029 (2.67%)	1,237,719 (2.32%)
halfy5	39,888,837 (90.37%)	3,011,769 (6.82%)	1,236,952 (2.8%)	1,113,677 (2.52%)
halfy6	57,074,467 (91.29%)	3,373,453 (5.4%)	2,070,155 (3.31%)	1,367,381 (2.19%)
halfy7	45,691,953 (90.64%)	2,815,511 (5.59%)	1,902,995 (3.78%)	1,175,948 (2.33%)
halfy8	45,469,814 (89.48%)	3,772,685 (7.42%)	1,571,241 (3.09%)	1,361,597 (2.68%)
halfy9	48,892,255 (90.04%)	3,840,448 (7.07%)	1,568,378 (2.89%)	1,378,863 (2.54%)
twoy1	49,000,231 (88.15%)	4,742,145 (8.53%)	1,845,531 (3.32%)	1,473,069 (2.65%)
twoy2	60,686,496 (90.68%)	4,316,500 (6.45%)	1,922,914 (2.87%)	1,566,625 (2.34%)
twoy3	59,759,533 (87.64%)	5,319,765 (7.8%)	3,111,788 (4.56%)	1,720,265 (2.52%)
twoy4	62,136,346 (90.15%)	4,816,569 (6.99%)	1,971,351 (2.86%)	1,546,058 (2.24%)
twoy5	42,535,369 (90.31%)	2,830,132 (6.01%)	1,735,956 (3.69%)	1,103,843 (2.34%)
twoy6	52,686,552 (89.25%)	4,531,664 (7.68%)	1,814,977 (3.07%)	1,437,006 (2.43%)
twoy7	64,821,544 (90.71%)	4,622,723 (6.47%)	2,015,562 (2.82%)	1,615,909 (2.26%)
twoy8	49,721,445 (89.6%)	3,498,469 (6.3%)	2,270,004 (4.09%)	1,323,921 (2.39%)
twoy9	52,374,518 (90.49%)	3,604,451 (6.23%)	1,898,604 (3.28%)	1,385,649 (2.39%)

Table S4. List of gene-specific primers for quantitative real-time PCR.

Gene Name		Primers	Product Length
LOC102172960 (CYP4B1)	F	GTGGACTGACCCTGAGGTCT	221
	R	TTGAGGGATCCGGGGAAAAC	
RRM2	F	GGAGAACGTGTGGTAGCCTT	150
	R	GTCACAGTGCAAACCCTCGT	
HACD1	F	TTGCCTTGCTTGAGATAGTCCA	178
	R	ATCTCTGTCACCGTCCATGC	
ACTC1	F	TGTATGCTTCTGGCCGTACC	199
	R	ATCTCTCGTTCAGCGGTGGT	
TOP2A	F	ATCTTCACAATGCTCAGCCCT	157
	R	TACCAATTCCTTCAGCGCCA	
ASB14	F	TACTGGGGAGTGGAGATCCTA	118
	R	TGTGGAATGCAGACAGATTTACTTT	
ANKRD33B	F	ATGCTAGCAGGGGCGGAT	164
	R	GGCAGCTCTGGCTTGTACTT	
UBQLN4	F	GGCACCCTTGATTACCCCTC	267
	R	GCCTCCGTAACAGGTTCTCC	
DRD1	F	ACCCCAAGGCAAGGGCTTTA	140
	R	ATTGTCGTCACATTCCGGGT	
RAD1	F	TCTATGGTCCCGGTCTCCAG	220
	R	CCGAGGGATGCTCCTTTGC	
GOSR2	F	ACGTCCAACACTTGCAGACT	123
	R	TCTTCTGGGTCCCGTTTGTG	
HSF4	F	GGAGCTGTCCCTGCCAAAG	183
	R	ACGGGCATCTCCTTTCTTCG	
GADPH	F	TGAAGGTCGGTGTGAACGGATTTGG	227
	R	ACGACATACTCAGCACCAGCATCAC	
